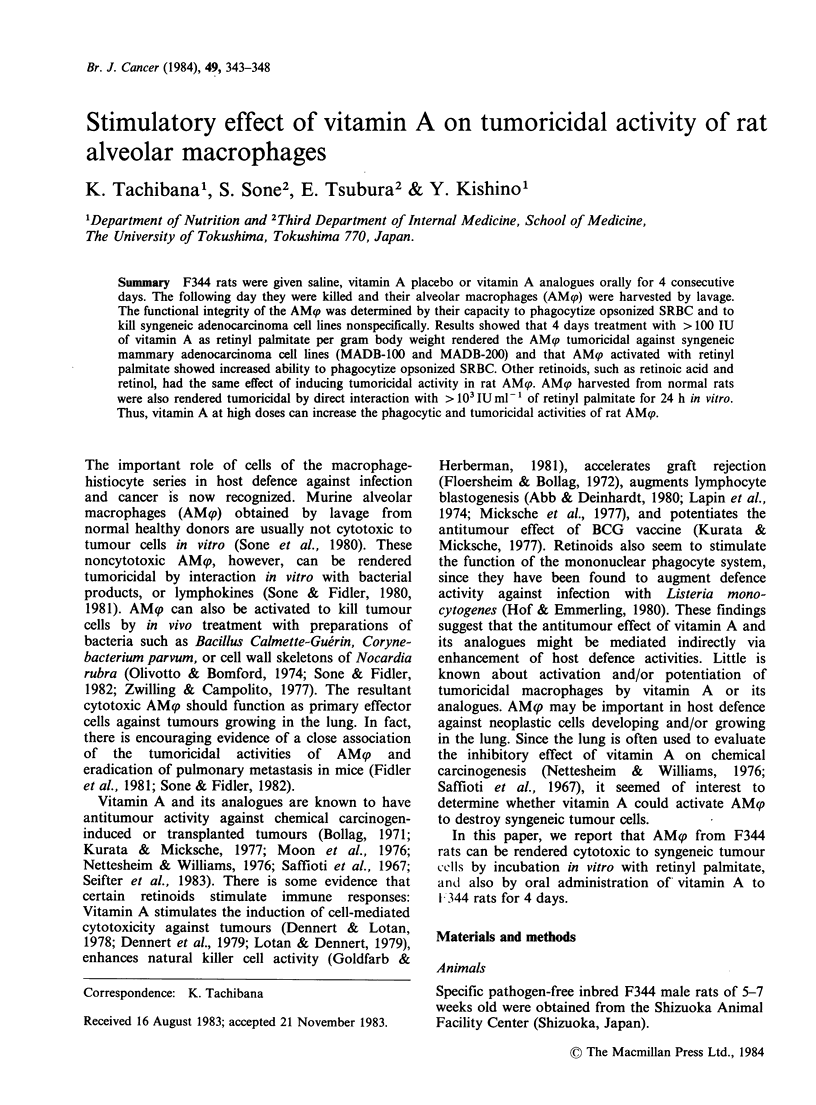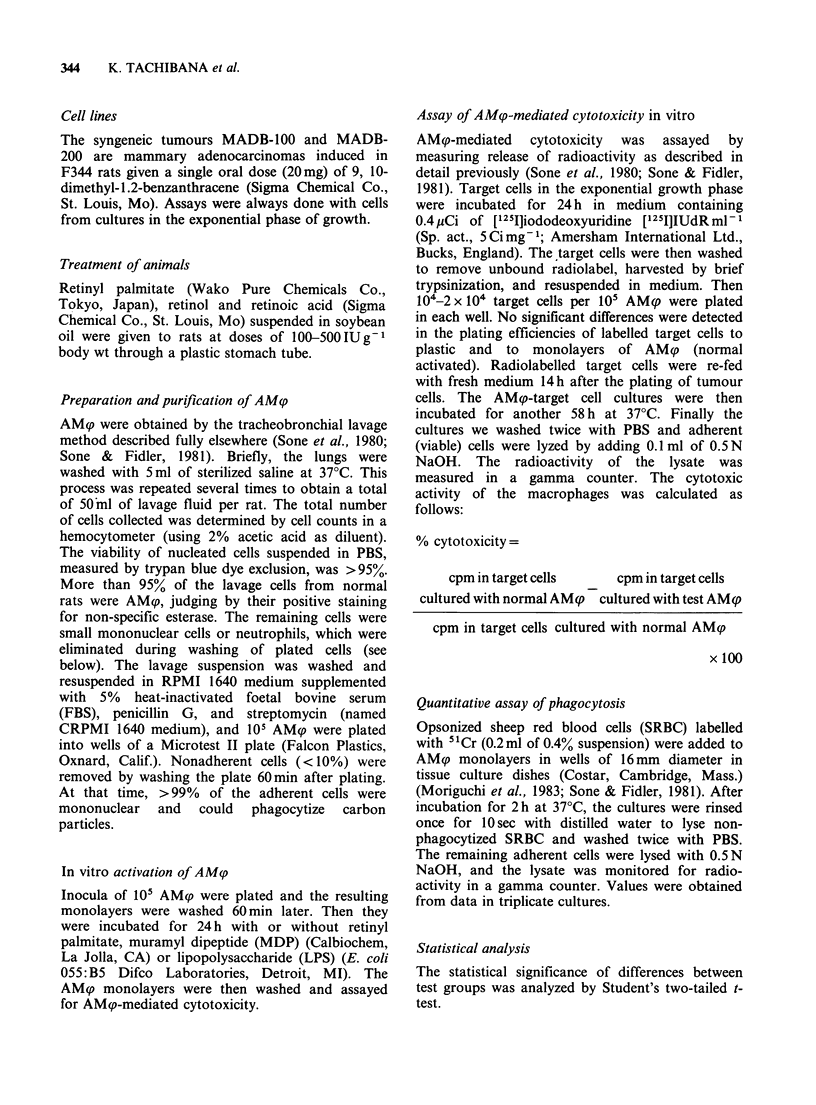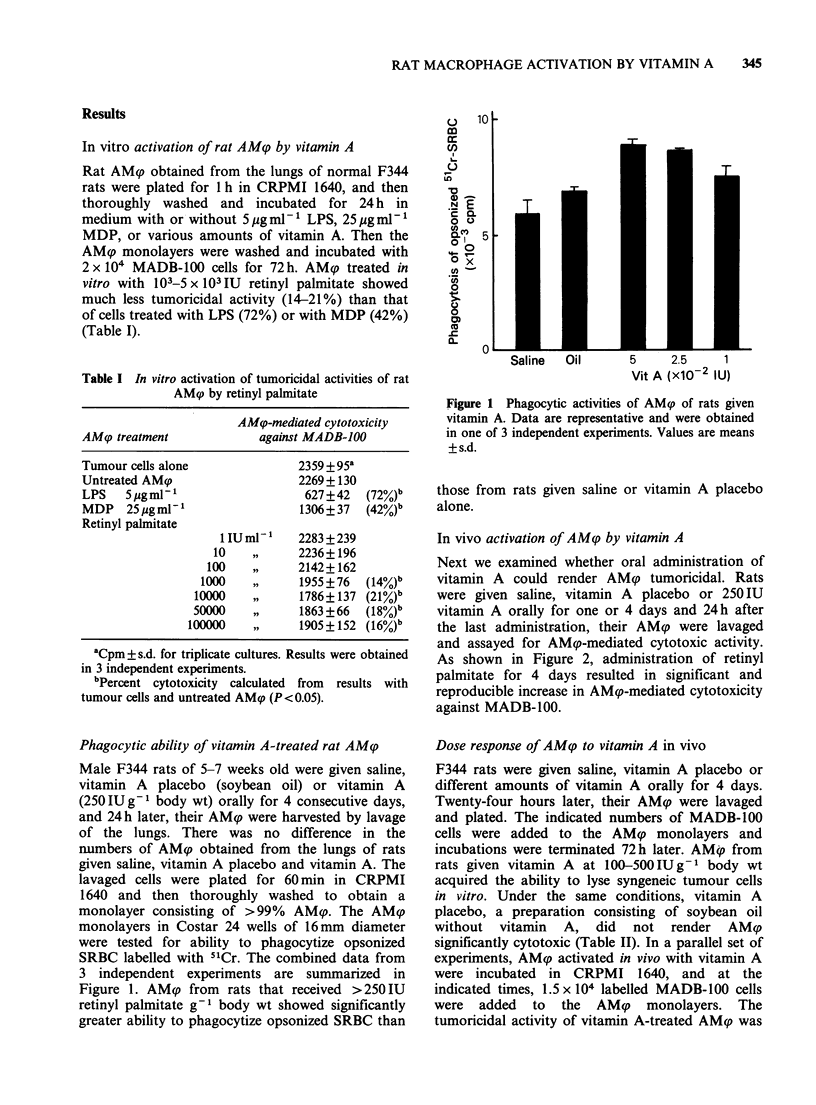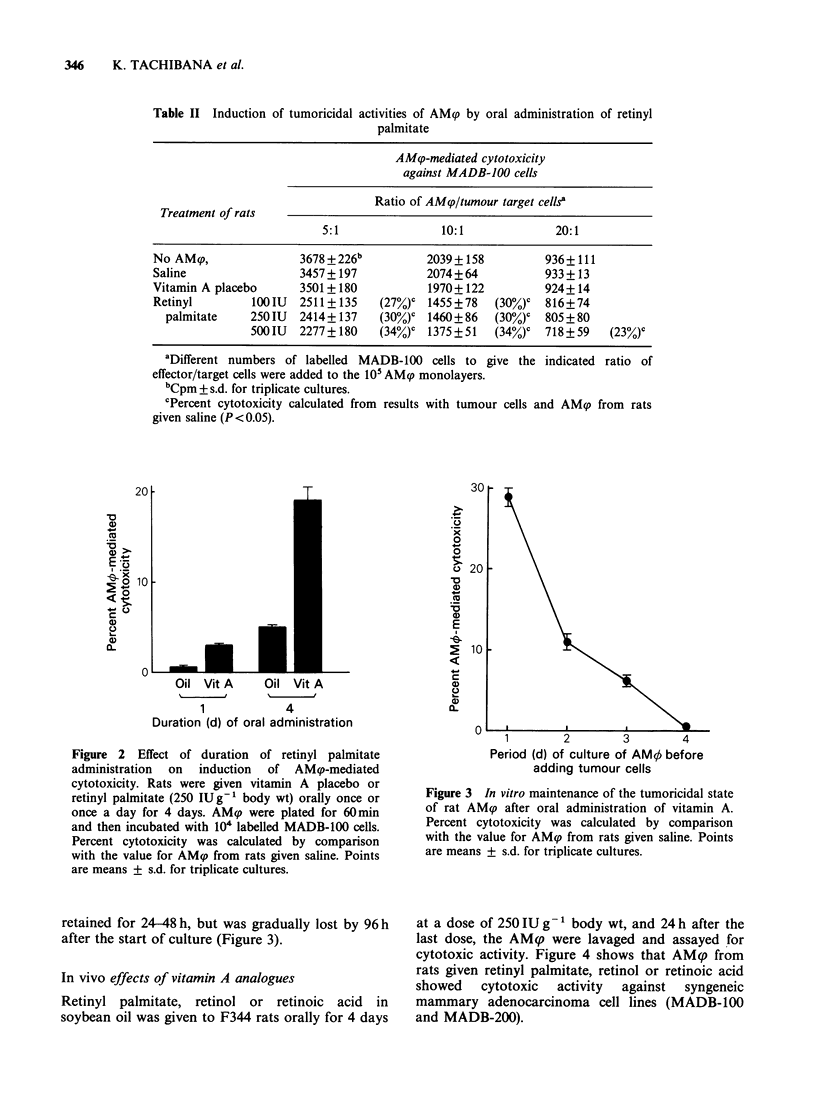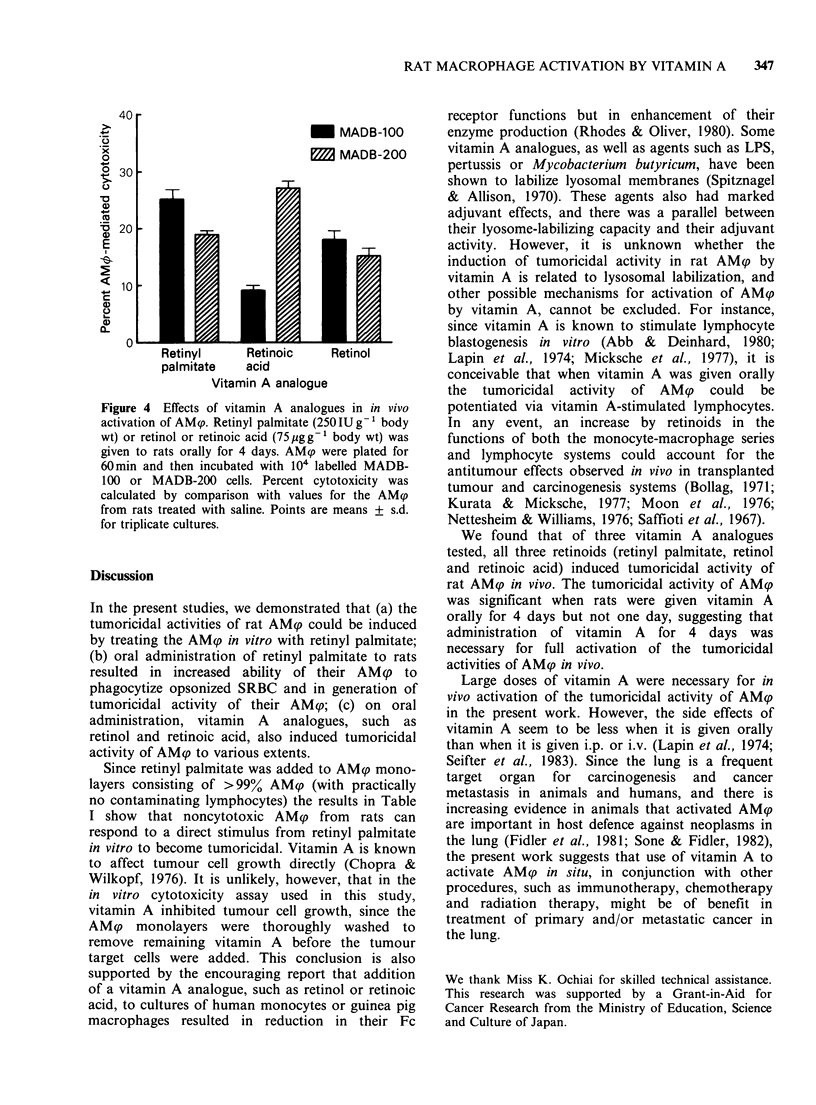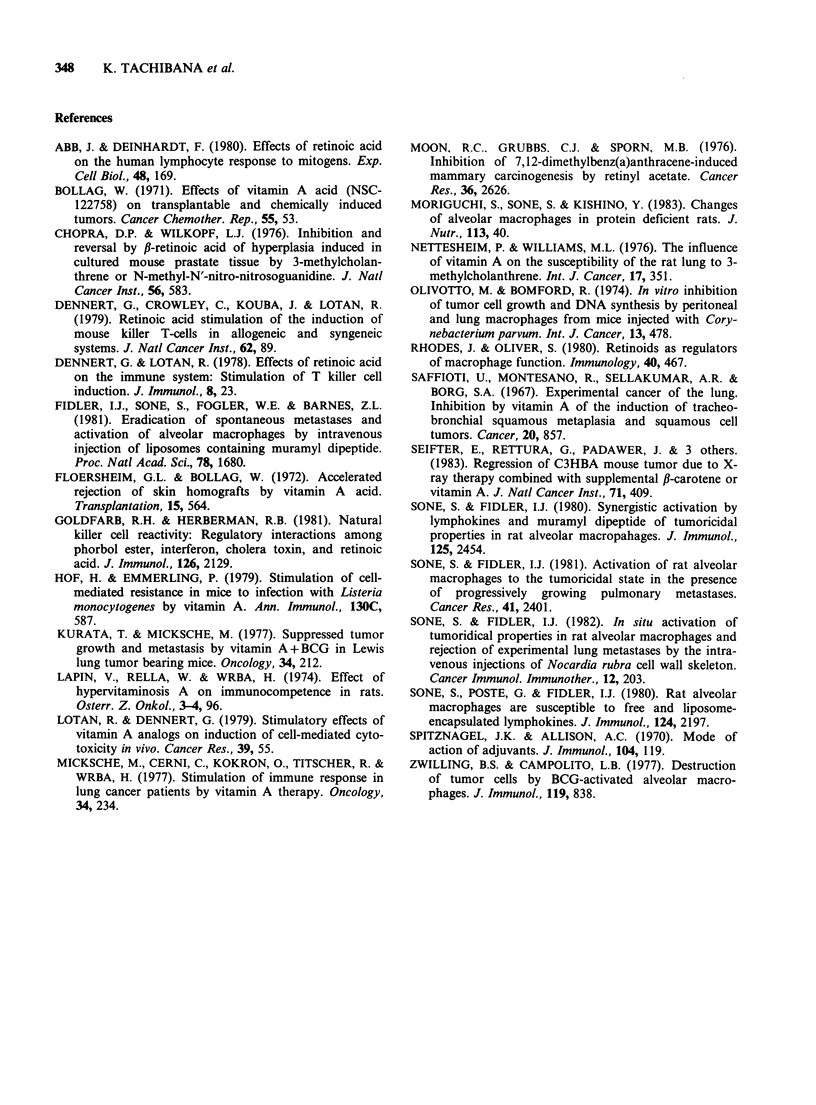# Stimulatory effect of vitamin A on tumoricidal activity of rat alveolar macrophages.

**DOI:** 10.1038/bjc.1984.54

**Published:** 1984-03

**Authors:** K. Tachibana, S. Sone, E. Tsubura, Y. Kishino

## Abstract

F344 rats were given saline, vitamin A placebo or vitamin A analogues orally for 4 consecutive days. The following day they were killed and their alveolar macrophages (AM phi) were harvested by lavage. The functional integrity of the AM phi was determined by their capacity to phagocytize opsonized SRBC and to kill syngeneic adenocarcinoma cell lines nonspecifically. Results showed that 4 days treatment with greater than 100 IU of vitamin A as retinyl palmitate per gram body weight rendered the AM phi tumoricidal against syngeneic mammary adenocarcinoma cell lines (MADB-100 and MADB-200) and that AM phi activated with retinyl palmitate showed increased ability to phagocytize opsonized SRBC. Other retinoids, such as retinoic acid and retinol, had the same effect of inducing tumoricidal activity in rat AM phi. AM phi harvested from normal rats were also rendered tumoricidal by direct interaction with greater than 10(3) IU ml-1 of retinyl palmitate for 24 h in vitro. Thus, vitamin A at high doses can increase the phagocytic and tumoricidal activities of rat AM phi.


*Br. J. Cancer* (1984), **49**, 343–348

### Stimulatory effect of vitamin A on tumoricidal activity of rat alveolar macrophages

K. Tachibana[1], S. Sone[2], E. Tsubura[2] & Y. Kishino[1]

[1]*Department of Nutrition and* [2]*Third Department of Internal Medicine, School of Medicine, The University of Tokushima, Tokushima 770, Japan.*

**Summary** F344 rats were given saline, vitamin A placebo or vitamin A analogues orally for 4 consecutive days. The following day they were killed and their alveolar macrophages (AMφ) were harvested by lavage. The functional integrity of the AMφ was determined by their capacity to phagocytize opsonized SRBC and to kill syngeneic adenocarcinoma cell lines nonspecifically. Results showed that 4 days treatment with >100 IU of vitamin A as retinyl palmitate per gram body weight rendered the AMφ tumoricidal against syngeneic mammary adenocarcinoma cell lines (MADB-100 and MADB-200) and that AMφ activated with retinyl palmitate showed increased ability to phagocytize opsonized SRBC. Other retinoids, such as retinoic acid and retinol, had the same effect of inducing tumoricidal activity in rat AMφ. AMφ harvested from normal rats were also rendered tumoricidal by direct interaction with $>10^3\,IU\,ml^{-1}$ of retinyl palmitate for 24 h *in vitro*. Thus, vitamin A at high doses can increase the phagocytic and tumoricidal activities of rat AMφ.

The important role of cells of the macrophage-histiocyte series in host defence against infection and cancer is now recognized. Murine alveolar macrophages (AMφ) obtained by lavage from normal healthy donors are usually not cytotoxic to tumour cells *in vitro* (Sone *et al.*, 1980). These noncytotoxic AMφ, however, can be rendered tumoricidal by interaction *in vitro* with bacterial products, or lymphokines (Sone & Fidler, 1980, 1981). AMφ can also be activated to kill tumour cells by *in vivo* treatment with preparations of bacteria such as *Bacillus Calmette-Guérin, Corynebacterium parvum,* or cell wall skeletons of *Nocardia rubra* (Olivotto & Bomford, 1974; Sone & Fidler, 1982; Zwilling & Campolito, 1977). The resultant cytotoxic AMφ should function as primary effector cells against tumours growing in the lung. In fact, there is encouraging evidence of a close association of the tumoricidal activities of AMφ and eradication of pulmonary metastasis in mice (Fidler *et al.*, 1981; Sone & Fidler, 1982).

Vitamin A and its analogues are known to have antitumour activity against chemical carcinogen-induced or transplanted tumours (Bollag, 1971; Kurata & Micksche, 1977; Moon *et al.*, 1976; Nettesheim & Williams, 1976; Saffioti *et al.*, 1967; Seifter *et al.*, 1983). There is some evidence that certain retinoids stimulate immune responses: Vitamin A stimulates the induction of cell-mediated cytotoxicity against tumours (Dennert & Lotan, 1978; Dennert *et al.*, 1979; Lotan & Dennert, 1979), enhances natural killer cell activity (Goldfarb & Herberman, 1981), accelerates graft rejection (Floersheim & Bollag, 1972), augments lymphocyte blastogenesis (Abb & Deinhardt, 1980; Lapin *et al.*, 1974; Micksche *et al.*, 1977), and potentiates the antitumour effect of BCG vaccine (Kurata & Micksche, 1977). Retinoids also seem to stimulate the function of the mononuclear phagocyte system, since they have been found to augment defence activity against infection with *Listeria monocytogenes* (Hof & Emmerling, 1980). These findings suggest that the antitumour effect of vitamin A and its analogues might be mediated indirectly via enhancement of host defence activities. Little is known about activation and/or potentiation of tumoricidal macrophages by vitamin A or its analogues. AMφ may be important in host defence against neoplastic cells developing and/or growing in the lung. Since the lung is often used to evaluate the inhibitory effect of vitamin A on chemical carcinogenesis (Nettesheim & Williams, 1976; Saffioti *et al.*, 1967), it seemed of interest to determine whether vitamin A could activate AMφ to destroy syngeneic tumour cells.

In this paper, we report that AMφ from F344 rats can be rendered cytotoxic to syngeneic tumour cells by incubation *in vitro* with retinyl palmitate, and also by oral administration of vitamin A to F344 rats for 4 days.

### Materials and methods

### *Animals*

Specific pathogen-free inbred F344 male rats of 5–7 weeks old were obtained from the Shizuoka Animal Facility Center (Shizuoka, Japan).

Correspondence: K. Tachibana

Received 16 August 1983; accepted 21 November 1983.

© The Macmillan Press Ltd., 1984

*Cell lines*

The syngeneic tumours MADB-100 and MADB-200 are mammary adenocarcinomas induced in F344 rats given a single oral dose (20 mg) of 9, 10-dimethyl-1.2-benzanthracene (Sigma Chemical Co., St. Louis, Mo). Assays were always done with cells from cultures in the exponential phase of growth.

*Treatment of animals*

Retinyl palmitate (Wako Pure Chemicals Co., Tokyo, Japan), retinol and retinoic acid (Sigma Chemical Co., St. Louis, Mo) suspended in soybean oil were given to rats at doses of 100–500 IU $g^{-1}$ body wt through a plastic stomach tube.

*Preparation and purification of AMφ*

AMφ were obtained by the tracheobronchial lavage method described fully elsewhere (Sone *et al.*, 1980; Sone & Fidler, 1981). Briefly, the lungs were washed with 5 ml of sterilized saline at 37°C. This process was repeated several times to obtain a total of 50 ml of lavage fluid per rat. The total number of cells collected was determined by cell counts in a hemocytometer (using 2% acetic acid as diluent). The viability of nucleated cells suspended in PBS, measured by trypan blue dye exclusion, was >95%. More than 95% of the lavage cells from normal rats were AMφ, judging by their positive staining for non-specific esterase. The remaining cells were small mononuclear cells or neutrophils, which were eliminated during washing of plated cells (see below). The lavage suspension was washed and resuspended in RPMI 1640 medium supplemented with 5% heat-inactivated foetal bovine serum (FBS), penicillin G, and streptomycin (named CRPMI 1640 medium), and $10^5$ AMφ were plated into wells of a Microtest II plate (Falcon Plastics, Oxnard, Calif.). Nonadherent cells (<10%) were removed by washing the plate 60 min after plating. At that time, >99% of the adherent cells were mononuclear and could phagocytize carbon particles.

In vitro *activation of AMφ*

Inocula of $10^5$ AMφ were plated and the resulting monolayers were washed 60 min later. Then they were incubated for 24 h with or without retinyl palmitate, muramyl dipeptide (MDP) (Calbiochem, La Jolla, CA) or lipopolysaccharide (LPS) (*E. coli* 055:B5 Difco Laboratories, Detroit, MI). The AMφ monolayers were then washed and assayed for AMφ-mediated cytotoxicity.

*Assay of AMφ-mediated cytotoxicity* in vitro

AMφ-mediated cytotoxicity was assayed by measuring release of radioactivity as described in detail previously (Sone *et al.*, 1980; Sone & Fidler, 1981). Target cells in the exponential growth phase were incubated for 24 h in medium containing 0.4 μCi of [$^{125}$I]iododeoxyuridine [$^{125}$I]IUdR $ml^{-1}$ (Sp. act., 5 Ci $mg^{-1}$; Amersham International Ltd., Bucks, England). The target cells were then washed to remove unbound radiolabel, harvested by brief trypsinization, and resuspended in medium. Then $10^4$–$2\times10^4$ target cells per $10^5$ AMφ were plated in each well. No significant differences were detected in the plating efficiencies of labelled target cells to plastic and to monolayers of AMφ (normal activated). Radiolabelled target cells were re-fed with fresh medium 14 h after the plating of tumour cells. The AMφ-target cell cultures were then incubated for another 58 h at 37°C. Finally the cultures we washed twice with PBS and adherent (viable) cells were lyzed by adding 0.1 ml of 0.5 N NaOH. The radioactivity of the lysate was measured in a gamma counter. The cytotoxic activity of the macrophages was calculated as follows:

$$\% \text{ cytotoxicity} = \frac{\begin{array}{c}\text{cpm in target cells}\\ \text{cultured with normal AM}\varphi\end{array} - \begin{array}{c}\text{cpm in target cells}\\ \text{cultured with test AM}\varphi\end{array}}{\text{cpm in target cells cultured with normal AM}\varphi} \times 100$$

*Quantitative assay of phagocytosis*

Opsonized sheep red blood cells (SRBC) labelled with $^{51}$Cr (0.2 ml of 0.4% suspension) were added to AMφ monolayers in wells of 16 mm diameter in tissue culture dishes (Costar, Cambridge, Mass.) (Moriguchi *et al.*, 1983; Sone & Fidler, 1981). After incubation for 2 h at 37°C, the cultures were rinsed once for 10 sec with distilled water to lyse non-phagocytized SRBC and washed twice with PBS. The remaining adherent cells were lysed with 0.5 N NaOH, and the lysate was monitored for radioactivity in a gamma counter. Values were obtained from data in triplicate cultures.

*Statistical analysis*

The statistical significance of differences between test groups was analyzed by Student's two-tailed *t*-test.

### Results

### In vitro *activation of rat AMφ by vitamin A*

Rat AMφ obtained from the lungs of normal F344 rats were plated for 1 h in CRPMI 1640, and then thoroughly washed and incubated for 24 h in medium with or without 5 μg ml$^{-1}$ LPS, 25 μg ml$^{-1}$ MDP, or various amounts of vitamin A. Then the AMφ monolayers were washed and incubated with $2 \times 10^4$ MADB-100 cells for 72 h. AMφ treated *in vitro* with $10^3$–$5 \times 10^3$ IU retinyl palmitate showed much less tumoricidal activity (14–21%) than that of cells treated with LPS (72%) or with MDP (42%) (Table I).

**Table I** *In vitro* activation of tumoricidal activities of rat AMφ by retinyl palmitate

| *AMφ treatment* | | *AMφ-mediated cytotoxicity against MADB-100* | |
|---|---|---|---|
| Tumour cells alone | | 2359±95[a] | |
| Untreated AMφ | | 2269±130 | |
| LPS 5 μg ml$^{-1}$ | | 627±42 | (72%)[b] |
| MDP 25 μg ml$^{-1}$ | | 1306±37 | (42%)[b] |
| Retinyl palmitate | | | |
| | 1 IU ml$^{-1}$ | 2283±239 | |
| | 10 " | 2236±196 | |
| | 100 " | 2142±162 | |
| | 1000 " | 1955±76 | (14%)[b] |
| | 10000 " | 1786±137 | (21%)[b] |
| | 50000 " | 1863±66 | (18%)[b] |
| | 100000 " | 1905±152 | (16%)[b] |

[a]Cpm±s.d. for triplicate cultures. Results were obtained in 3 independent experiments.

[b]Percent cytotoxicity calculated from results with tumour cells and untreated AMφ ($P<0.05$).

### *Phagocytic ability of vitamin A-treated rat AMφ*

Male F344 rats of 5–7 weeks old were given saline, vitamin A placebo (soybean oil) or vitamin A (250 IU g$^{-1}$ body wt) orally for 4 consecutive days, and 24 h later, their AMφ were harvested by lavage of the lungs. There was no difference in the numbers of AMφ obtained from the lungs of rats given saline, vitamin A placebo and vitamin A. The lavaged cells were plated for 60 min in CRPMI 1640 and then thoroughly washed to obtain a monolayer consisting of >99% AMφ. The AMφ monolayers in Costar 24 wells of 16 mm diameter were tested for ability to phagocytize opsonized SRBC labelled with $^{51}Cr$. The combined data from 3 independent experiments are summarized in Figure 1. AMφ from rats that received >250 IU retinyl palmitate g$^{-1}$ body wt showed significantly greater ability to phagocytize opsonized SRBC than those from rats given saline or vitamin A placebo alone.

**Figure 1** Phagocytic activities of AMφ of rats given vitamin A. Data are representative and were obtained in one of 3 independent experiments. Values are means ±s.d.

### In vivo *activation of AMφ by vitamin A*

Next we examined whether oral administration of vitamin A could render AMφ tumoricidal. Rats were given saline, vitamin A placebo or 250 IU vitamin A orally for one or 4 days and 24 h after the last administration, their AMφ were lavaged and assayed for AMφ-mediated cytotoxic activity. As shown in Figure 2, administration of retinyl palmitate for 4 days resulted in significant and reproducible increase in AMφ-mediated cytotoxicity against MADB-100.

### *Dose response of AMφ to vitamin A* in vivo

F344 rats were given saline, vitamin A placebo or different amounts of vitamin A orally for 4 days. Twenty-four hours later, their AMφ were lavaged and plated. The indicated numbers of MADB-100 cells were added to the AMφ monolayers and incubations were terminated 72 h later. AMφ from rats given vitamin A at 100–500 IU g$^{-1}$ body wt acquired the ability to lyse syngeneic tumour cells *in vitro*. Under the same conditions, vitamin A placebo, a preparation consisting of soybean oil without vitamin A, did not render AMφ significantly cytotoxic (Table II). In a parallel set of experiments, AMφ activated *in vivo* with vitamin A were incubated in CRPMI 1640, and at the indicated times, $1.5 \times 10^4$ labelled MADB-100 cells were added to the AMφ monolayers. The tumoricidal activity of vitamin A-treated AMφ was

**Table II** Induction of tumoricidal activities of AMφ by oral administration of retinyl palmitate

| Treatment of rats | | AMφ-mediated cytotoxicity against MADB-100 cells: Ratio of AMφ/tumour target cells[a] | | | | | |
|---|---|---|---|---|---|---|---|
| | | 5:1 | | 10:1 | | 20:1 | |
| No AMφ, | | 3678±226[b] | | 2039±158 | | 936±111 | |
| Saline | | 3457±197 | | 2074±64 | | 933±13 | |
| Vitamin A placebo | | 3501±180 | | 1970±122 | | 924±14 | |
| Retinyl | 100 IU | 2511±135 | (27%)[c] | 1455±78 | (30%)[c] | 816±74 | |
| palmitate | 250 IU | 2414±137 | (30%)[c] | 1460±86 | (30%)[c] | 805±80 | |
| | 500 IU | 2277±180 | (34%)[c] | 1375±51 | (34%)[c] | 718±59 | (23%)[c] |

[a]Different numbers of labelled MADB-100 cells to give the indicated ratio of effector/target cells were added to the $10^5$ AMφ monolayers.

[b]Cpm ± s.d. for triplicate cultures.

[c]Percent cytotoxicity calculated from results with tumour cells and AMφ from rats given saline ($P<0.05$).

**Figure 2** Effect of duration of retinyl palmitate administration on induction of AMφ-mediated cytotoxicity. Rats were given vitamin A placebo or retinyl palmitate (250 IU $g^{-1}$ body wt) orally once or once a day for 4 days. AMφ were plated for 60 min and then incubated with $10^4$ labelled MADB-100 cells. Percent cytotoxicity was calculated by comparison with the value for AMφ from rats given saline. Points are means ± s.d. for triplicate cultures.

**Figure 3** *In vitro* maintenance of the tumoricidal state of rat AMφ after oral administration of vitamin A. Percent cytotoxicity was calculated by comparison with the value for AMφ from rats given saline. Points are means ± s.d. for triplicate cultures.

retained for 24–48 h, but was gradually lost by 96 h after the start of culture (Figure 3).

### *In vivo effects of vitamin A analogues*

Retinyl palmitate, retinol or retinoic acid in soybean oil was given to F344 rats orally for 4 days at a dose of 250 IU $g^{-1}$ body wt, and 24 h after the last dose, the AMφ were lavaged and assayed for cytotoxic activity. Figure 4 shows that AMφ from rats given retinyl palmitate, retinol or retinoic acid showed cytotoxic activity against syngeneic mammary adenocarcinoma cell lines (MADB-100 and MADB-200).

**Figure 4** Effects of vitamin A analogues in *in vivo* activation of AMφ. Retinyl palmitate (250 IU $g^{-1}$ body wt) or retinol or retinoic acid (75 $\mu g\, g^{-1}$ body wt) was given to rats orally for 4 days. AMφ were plated for 60 min and then incubated with $10^4$ labelled MADB-100 or MADB-200 cells. Percent cytotoxicity was calculated by comparison with values for the AMφ from rats treated with saline. Points are means ± s.d. for triplicate cultures.

### Discussion

In the present studies, we demonstrated that (a) the tumoricidal activities of rat AMφ could be induced by treating the AMφ *in vitro* with retinyl palmitate; (b) oral administration of retinyl palmitate to rats resulted in increased ability of their AMφ to phagocytize opsonized SRBC and in generation of tumoricidal activity of their AMφ; (c) on oral administration, vitamin A analogues, such as retinol and retinoic acid, also induced tumoricidal activity of AMφ to various extents.

Since retinyl palmitate was added to AMφ monolayers consisting of >99% AMφ (with practically no contaminating lymphocytes) the results in Table I show that noncytotoxic AMφ from rats can respond to a direct stimulus from retinyl palmitate *in vitro* to become tumoricidal. Vitamin A is known to affect tumour cell growth directly (Chopra & Wilkopf, 1976). It is unlikely, however, that in the *in vitro* cytotoxicity assay used in this study, vitamin A inhibited tumour cell growth, since the AMφ monolayers were thoroughly washed to remove remaining vitamin A before the tumour target cells were added. This conclusion is also supported by the encouraging report that addition of a vitamin A analogue, such as retinol or retinoic acid, to cultures of human monocytes or guinea pig macrophages resulted in reduction in their Fc receptor functions but in enhancement of their enzyme production (Rhodes & Oliver, 1980). Some vitamin A analogues, as well as agents such as LPS, pertussis or *Mycobacterium butyricum*, have been shown to labilize lyosomal membranes (Spitznagel & Allison, 1970). These agents also had marked adjuvant effects, and there was a parallel between their lyosome-labilizing capacity and their adjuvant activity. However, it is unknown whether the induction of tumoricidal activity in rat AMφ by vitamin A is related to lysosomal labilization, and other possible mechanisms for activation of AMφ by vitamin A, cannot be excluded. For instance, since vitamin A is known to stimulate lymphocyte blastogenesis *in vitro* (Abb & Deinhard, 1980; Lapin *et al.*, 1974; Micksche *et al.*, 1977), it is conceivable that when vitamin A was given orally the tumoricidal activity of AMφ could be potentiated via vitamin A-stimulated lymphocytes. In any event, an increase by retinoids in the functions of both the monocyte-macrophage series and lymphocyte systems could account for the antitumour effects observed *in vivo* in transplanted tumour and carcinogenesis systems (Bollag, 1971; Kurata & Micksche, 1977; Moon *et al.*, 1976; Nettesheim & Williams, 1976; Saffioti *et al.*, 1967).

We found that of three vitamin A analogues tested, all three retinoids (retinyl palmitate, retinol and retinoic acid) induced tumoricidal activity of rat AMφ *in vivo*. The tumoricidal activity of AMφ was significant when rats were given vitamin A orally for 4 days but not one day, suggesting that administration of vitamin A for 4 days was necessary for full activation of the tumoricidal activities of AMφ *in vivo*.

Large doses of vitamin A were necessary for *in vivo* activation of the tumoricidal activity of AMφ in the present work. However, the side effects of vitamin A seem to be less when it is given orally than when it is given i.p. or i.v. (Lapin *et al.*, 1974; Seifter *et al.*, 1983). Since the lung is a frequent target organ for carcinogenesis and cancer metastasis in animals and humans, and there is increasing evidence in animals that activated AMφ are important in host defence against neoplasms in the lung (Fidler *et al.*, 1981; Sone & Fidler, 1982), the present work suggests that use of vitamin A to activate AMφ *in situ*, in conjunction with other procedures, such as immunotherapy, chemotherapy and radiation therapy, might be of benefit in treatment of primary and/or metastatic cancer in the lung.

We thank Miss K. Ochiai for skilled technical assistance. This research was supported by a Grant-in-Aid for Cancer Research from the Ministry of Education, Science and Culture of Japan.

### References

ABB, J. & DEINHARDT, F. (1980). Effects of retinoic acid on the human lymphocyte response to mitogens. *Exp. Cell Biol.*, **48**, 169.

BOLLAG, W. (1971). Effects of vitamin A acid (NSC-122758) on transplantable and chemically induced tumors. *Cancer Chemother. Rep.*, **55**, 53.

CHOPRA, D.P. & WILKOPF, L.J. (1976). Inhibition and reversal by β-retinoic acid of hyperplasia induced in cultured mouse prastate tissue by 3-methylcholanthrene or N-methyl-N′-nitro-nitrosoguanidine. *J. Natl Cancer Inst.*, **56**, 583.

DENNERT, G., CROWLEY, C., KOUBA, J. & LOTAN, R. (1979). Retinoic acid stimulation of the induction of mouse killer T-cells in allogeneic and syngeneic systems. *J. Natl Cancer Inst.*, **62**, 89.

DENNERT, G. & LOTAN, R. (1978). Effects of retinoic acid on the immune system: Stimulation of T killer cell induction. *J. Immunol.*, **8**, 23.

FIDLER, I.J., SONE, S., FOGLER, W.E. & BARNES, Z.L. (1981). Eradication of spontaneous metastases and activation of alveolar macrophages by intravenous injection of liposomes containing muramyl dipeptide. *Proc. Natl Acad. Sci.*, **78**, 1680.

FLOERSHEIM, G.L. & BOLLAG, W. (1972). Accelerated rejection of skin homografts by vitamin A acid. *Transplantation*, **15**, 564.

GOLDFARB, R.H. & HERBERMAN, R.B. (1981). Natural killer cell reactivity: Regulatory interactions among phorbol ester, interferon, cholera toxin, and retinoic acid. *J. Immunol.*, **126**, 2129.

HOF, H. & EMMERLING, P. (1979). Stimulation of cell-mediated resistance in mice to infection with *Listeria monocytogenes* by vitamin A. *Ann. Immunol.*, **130C**, 587.

KURATA, T. & MICKSCHE, M. (1977). Suppressed tumor growth and metastasis by vitamin A+BCG in Lewis lung tumor bearing mice. *Oncology*, **34**, 212.

LAPIN, V., RELLA, W. & WRBA, H. (1974). Effect of hypervitaminosis A on immunocompetence in rats. *Osterr. Z. Onkol.*, **3–4**, 96.

LOTAN, R. & DENNERT, G. (1979). Stimulatory effects of vitamin A analogs on induction of cell-mediated cytotoxicity *in vivo*. *Cancer Res.*, **39**, 55.

MICKSCHE, M., CERNI, C., KOKRON, O., TITSCHER, R. & WRBA, H. (1977). Stimulation of immune response in lung cancer patients by vitamin A therapy. *Oncology*, **34**, 234.

MOON, R.C., GRUBBS, C.J. & SPORN, M.B. (1976). Inhibition of 7,12-dimethylbenz(a)anthracene-induced mammary carcinogenesis by retinyl acetate. *Cancer Res.*, **36**, 2626.

MORIGUCHI, S., SONE, S. & KISHINO, Y. (1983). Changes of alveolar macrophages in protein deficient rats. *J. Nutr.*, **113**, 40.

NETTESHEIM, P. & WILLIAMS, M.L. (1976). The influence of vitamin A on the susceptibility of the rat lung to 3-methylcholanthrene. *Int. J. Cancer*, **17**, 351.

OLIVOTTO, M. & BOMFORD, R. (1974). *In vitro* inhibition of tumor cell growth and DNA synthesis by peritoneal and lung macrophages from mice injected with *Corynebacterium parvum*. *Int. J. Cancer*, **13**, 478.

RHODES, J. & OLIVER, S. (1980). Retinoids as regulators of macrophage function. *Immunology*, **40**, 467.

SAFFIOTI, U., MONTESANO, R., SELLAKUMAR, A.R. & BORG, S.A. (1967). Experimental cancer of the lung. Inhibition by vitamin A of the induction of tracheobronchial squamous metaplasia and squamous cell tumors. *Cancer*, **20**, 857.

SEIFTER, E., RETTURA, G., PADAWER, J. & 3 others. (1983). Regression of C3HBA mouse tumor due to X-ray therapy combined with supplemental β-carotene or vitamin A. *J. Natl Cancer Inst.*, **71**, 409.

SONE, S. & FIDLER, I.J. (1980). Synergistic activation by lymphokines and muramyl dipeptide of tumoricidal properties in rat alveolar macropahages. *J. Immunol.*, **125**, 2454.

SONE, S. & FIDLER, I.J. (1981). Activation of rat alveolar macrophages to the tumoricidal state in the presence of progressively growing pulmonary metastases. *Cancer Res.*, **41**, 2401.

SONE, S. & FIDLER, I.J. (1982). *In situ* activation of tumoridical properties in rat alveolar macrophages and rejection of experimental lung metastases by the intravenous injections of *Nocardia rubra* cell wall skeleton. *Cancer Immunol. Immunother.*, **12**, 203.

SONE, S., POSTE, G. & FIDLER, I.J. (1980). Rat alveolar macrophages are susceptible to free and liposome-encapsulated lymphokines. *J. Immunol.*, **124**, 2197.

SPITZNAGEL, J.K. & ALLISON, A.C. (1970). Mode of action of adjuvants. *J. Immunol.*, **104**, 119.

ZWILLING, B.S. & CAMPOLITO, L.B. (1977). Destruction of tumor cells by BCG-activated alveolar macrophages. *J. Immunol.*, **119**, 838.